# Measuring treatment effects on dual-task performance: a framework for research and clinical practice

**DOI:** 10.3389/fnhum.2015.00225

**Published:** 2015-04-28

**Authors:** Prudence Plummer, Gail Eskes

**Affiliations:** ^1^Division of Physical Therapy, University of North Carolina at Chapel HillChapel Hill, NC, USA; ^2^Department of Psychiatry, Dalhousie University, HalifaxNova Scotia, Canada

**Keywords:** cognitive-motor interference, attention allocation, task prioritization, gait rehabilitation, physical therapy, cognition, capacity sharing

## Abstract

The relevance of dual-task walking to everyday ambulation is widely acknowledged, and numerous studies have demonstrated that dual-task interference can significantly impact recovery of functional walking in people with neurological disorders. The magnitude and direction of dual-task interference is influenced by the interaction between the two tasks, including how individuals spontaneously prioritize their attention. Therefore, to accurately interpret and characterize dual-task interference and identify changes over time, it is imperative to evaluate single and dual-task performance in both tasks, as well as the tasks relative to each other. Yet, reciprocal dual-task effects (DTE) are frequently ignored. The purpose of this perspective paper is to present a framework for measuring treatment effects on dual-task interference, specifically taking into account the interactions between the two tasks and how this can provide information on whether overall dual-task capacity has improved or a different attentional strategy has been adopted. In discussing the clinical implications of using this framework, we provide specific examples of using this method and provide some explicit recommendations for research and clinical practice.

## Dual-Task Interference

In everyday life, we rarely perform only one task at a time. Rather, our day-to-day activities frequently involve the simultaneous performance of two or more tasks, such as walking and talking, or walking while searching for something in a pocket, referred to as *dual-tasking*. Given that attention is a limited resource, dividing attention between two concurrent tasks can result in a decrement in performance in one or both of the tasks, relative to when each task is performed alone (i.e., without competing attentional demands), especially when the attentional demands of one of the tasks is high (Abenethy, 1988). The relative change in performance associated with dual-tasking is referred to as *dual-task interference* or the *dual-task effect (DTE)*. Individuals with neurological deficits may be particularly susceptible to dual-task interference, because the relative increase in attentional demands to control motor performance means that there are fewer attentional resources available for simultaneous performance of secondary tasks. Given the importance of community ambulation for participation and quality of life (Aström et al., 1993; Bond et al., 1995; Roos et al., 2012), and the relevance of dual-task walking to everyday community ambulation, it is not surprising that dual-task walking performance is becoming a rehabilitation outcome of focus among individuals with neurological disorders (Brauer et al., 2011; Kelly et al., 2012; Plummer-D’Amato et al., 2012). Unfortunately, dual-task interference is often measured inadequately in rehabilitation studies, thereby limiting what we currently know about the effect of treatment of dual-task performance.

The purpose of this perspective paper is to present a framework for measuring treatment effects on dual-task interference, specifically taking into account the interactions between the two tasks as an indicator of task abilities. In presenting this framework, we focus on a cognitive-motor dual-task paradigm during walking as a model for discussion, but the principles can be applied to any dual-task combination in any population of interest. Thus, the paradigm we propose is process-specific, not task- or disease-specific. We provide a brief overview of the mechanisms of dual-task interference and discuss the possible brain-behavior relationships involved in dual-task performance. Examples from rehabilitation research are presented to illustrate how traditional methods of assessing dual-task performance can result in misleading interpretation of rehabilitation-related changes in dual-task interference, highlighting the need for a more comprehensive assessment model.

## Theoretical Accounts of Dual-Task Interference

The question of how dual-task interference occurs remains a question of ongoing debate. However, there are two major competing, but related, theories regarding the mechanisms underlying dual-task interference. The *serial bottleneck model* of dual-task interference proposes that dual-task costs arise because only one information processing operation can proceed at a time (Pashler, 1984, 1994). In contrast, the *capacity sharing model* argues that processing of multiple tasks can proceed in parallel, but the central processing capacity to do so is limited (Tombu and Jolicoeur, 2003). When there is limited capacity to perform two operations simultaneously, capacity may be allocated to one task over the other (Tombu and Jolicoeur, 2003, 2005). Capacity allocation may be voluntary or influenced by characteristics of the tasks (Tombu and Jolicoeur, 2003). In this regard, the capacity sharing model considers serial processing, such as that explained by the bottleneck model, to be an adopted strategy (Meyer and Kieras, 1997; Tombu and Jolicoeur, 2003; Miller et al., 2009). The idea of selecting a particular strategy for dual-task performance is consistent with the *model of task prioritization* (Yogev-Seligmann et al., 2012), which suggests that when there is competition for attentional resources, the person must decide how to prioritize the two tasks, and that this self-selected strategy of task prioritization is determined by factors that minimize danger and maximize pleasure. Thus, factors such as an individual’s physical capacity to respond to a postural threat (termed *postural reserve*) and one’s ability to recognize potential hazards in the environment and the situation primarily impact how attention (capacity) is allocated. Consequently, this decision-making process influences the magnitude and direction of dual-task interference in each task. For example, if an individual must pay more attention to posture and stability to avoid falling, then performance on the cognitive task may be compromised; this may not be the case in a less threatening task/environment or for a person with greater postural reserve.

## Brain-Behavior Relationships in Dual-Tasking

Brain-behavior relationships in dual-tasking are largely unknown. However, recent imaging research has suggested that lateral prefrontal cortical structures are recruited when dual-tasking involves more serial response selection, whereas striatal structures of the basal ganglia are recruited when there is a more parallel response selection process (Yildiz and Beste, 2014). Yogev-Seligmann and colleagues (Yogev-Seligmann et al., 2012) have also recognized the role of the basal ganglia in their model of prioritization for dual-task interference. They propose that habitual responses (i.e., well-practiced and more automated tasks that require relatively little attentional resources), which are mediated by the basal ganglia, dominate in situations where one must act quickly and that habitual responses are characterized by parallel processing. Conversely, goal-directed behavior is more characterized by serial processing (Yogev-Seligmann et al., 2012). Because individuals with Parkinson’s disease experience neurodegeneration affecting basal ganglia structures responsible for habitual responses, they rely more heavily on goal-directed responses. Thus, in people with Parkinson’s disease, previously habitual (automatic) responses, such as gait, demand attentional resources making these individuals more prone to dual-task interference (Yogev-Seligmann et al., 2012). This explanation is consistent with the recent findings that serial response selection is mediated by prefrontal cortical mechanisms while parallel response selection is mediated via striatal mechanisms (Yildiz and Beste, 2014). While this research suggests one approach to defining brain-behavior models associated with dual-task interference, it is important to acknowledge that the interaction between brain structure (or lesion location) and behavioral outcome may be influenced by the type of tasks involved, as well as general psychological or physical factors, such as motivation, anxiety or fatiguability (Godefroy et al., 1998). Moreover, as brain-behavior models of dual-task interference develop further, they will also need to consider the networks involved in attention allocation, which underlie dual-task interactions according to the capacity sharing/prioritization models of dual-task interference.

## Characterizing and Measuring Dual-Task Interference

Dual-task interference is quantified by calculating a DTE for each of the two tasks. The traditional formula for evaluating the DTE on a particular outcome of interest (e.g., gait speed or accuracy), is (Kelly et al., 2010):

$$DTE\left(\%\right)=\frac{\left(\text{dual task gait speed − single task gait speed}\right)}{\text{single task gait speed}}\times 100\%$$

For variables in which higher values indicate worse (instead of better) performance (e.g., reaction time [RT]), a negative sign is inserted into the formula as follows:

$$DTE\left(\%\right)=\frac{-\left(\text{dual task RT − single task RT}\right)}{\text{single task RT}}\times 100\%$$

Therefore, by convention, negative DTE values indicate that performance deteriorated in the dual-task relative to the single-task (i.e., dual-task cost), whereas positive DTE values indicate a relative improvement in performance in the dual-task (i.e., dual-task benefit).

In assessing dual-task interference an important, yet often overlooked, point is that dual-task interference encompasses the DTE of *both* tasks. The importance of this is illustrated by considering that a dual-task decline in gait speed may occur with (1) a reciprocal dual-task decline in the cognitive task (i.e., mutual interference); or (2) no change in cognitive-task performance relative to single-task performance (i.e., motor interference with no cognitive interference); or (3) an improvement in cognitive task performance relative to single-task (i.e., motor interference with cognitive benefit) (Plummer et al., 2013). If only the DTE on gait were measured, ignoring the DTE on the cognitive task, then it would not be possible to differentiate these three scenarios from each other—they all would simply show “dual-task interference on gait.” Yet, these three situations display very different patterns (and possibly different levels of severity) of dual-task interference with different interpretations.

Mutual interference suggests that there are inadequate attentional resources (i.e., attentional demands exceed total capacity) to maintain single-task-level performance in each task when performed together, and therefore both deteriorate (although the two tasks may not necessarily deteriorate to the same degree). Interference in the motor task but not the cognitive task also suggests inadequate attentional resources, with the key difference from the previous scenario being apparent prioritization of the cognitive task such that only the gait task deteriorates while cognitive performance is maintained. That is, there is capacity sharing with primary allocation of capacity to the cognitive task. The third scenario suggests a trade-off in attentional resources, such that improved performance in the cognitive task occurred at a cost to gait. The latter could be considered a less severe form of dual-task interference on gait, since it suggests that the dual-task decline in gait occurred not because there were insufficient resources, but, rather, because resources were over-allocated to the cognitive task. As illustrated by these examples, to accurately interpret dual-task interference it is critical that performance on both tasks is measured in single and dual-task conditions so that relative DTE can be examined and the attentional strategy and overall dual-task performance better understood. Unfortunately, most studies usually report dual-task interference as the DTE on only the gait (motor) task. Indeed, many studies do not even measure performance of the cognitive task in dual-task conditions, let alone assess it in single-task conditions in order to quantify the DTE (Plummer et al., 2013, 2015).

Consideration of the interaction between the two tasks and the attention strategy is particularly important when evaluating dual-task performance before and after rehabilitation, since measuring only the DTE on gait can result in misleading conclusions about the effects of treatment. For example, a person may demonstrate reduced dual-task costs on gait after an intervention, which, if considered in isolation, would lead one to conclude that dual-task performance has improved. However, if the reduction in dual-task costs on gait is accompanied by a reciprocal increase in dual-task costs on the cognitive task, then the overall DTE has not improved. Rather, the person has simply used a different strategy to perform the dual-task at the post assessment. This example underscores how an incomplete assessment of dual-task performance can provide misleading information about treatment effects on dual-task interference. A complete assessment of dual-task performance requires single and dual-task assessment of both tasks before and after rehabilitation.

## Framework for Measuring Treatment Effects on Dual-Task Interference

**Figure 1:**
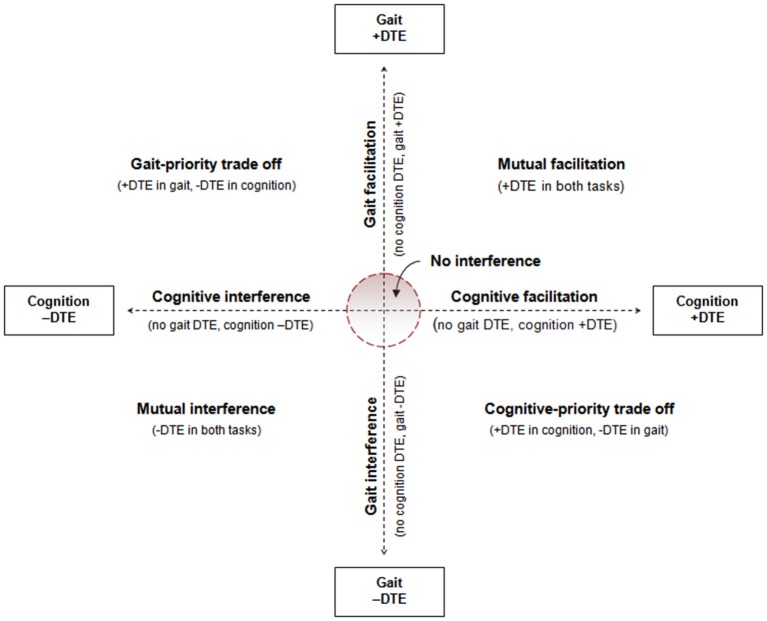
**Illustration of conceptual model for characterizing patterns of cognitive-motor dual-task interference**. Figure is from Plummer et al. (2014), and adapted from conceptual framework of Plummer et al. (2013).

We have previously described a conceptual framework for classifying patterns of dual-task interference (Plummer et al., 2013) illustrated in Figure 1. The purpose of this paper is to build on that framework to facilitate assessment and measurement of dual-task interference patterns, especially for evaluating change in dual-task interference over time. The pattern of dual-task interference can be classified by plotting the gait and cognitive DTE against one another. The “no interference” region is arbitrarily depicted in Figure 1. The boundaries of this region should be determined by the minimal clinically important difference (MCID) values for DTE. MCID values for DTE are currently not known. Clinically important values of DTE may differ between gait DTE and cognitive DTE, and will most likely differ based on a person’s absolute measures (e.g., single-task or dual-task gait speed), as we will explain below.

**Figure 2:**
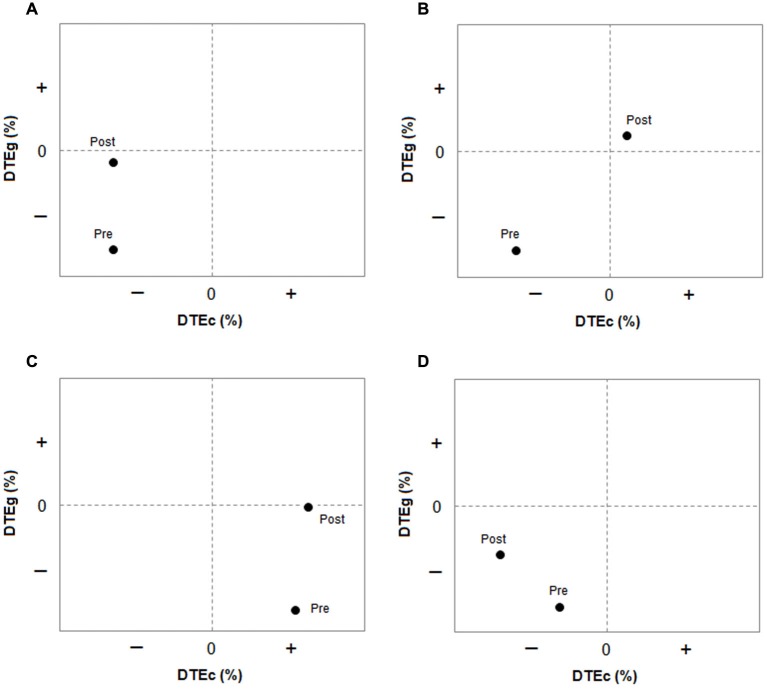
**Plots showing interaction between two tasks and the patterns of dual-task interference pre and post intervention.** DTE refers to Dual-task effect. DTEg is relative DTE on gait measure; DTEc is relative dual-task effect on cognitive measure. **(A)** Pre = mutual interference, post = improved gait interference (no change in cognitive interference); **(B)** Pre = mutual interference, post = improved gait interference (with improved cognitive interference); **(C)** Pre = gait interference with cognitive-priority trade off, post = improved gait interference (no change in cognitive DTE); **(D)** Pre = mutual interference, post = improved gait interference at cost to cognitive interference (worsened), therefore, no improvement but a change in strategy for dual-task performance. Data are hypothetical.

Although it is theoretically possible that individuals may present with any of the possible patterns of dual-task interference shown in Figure 1, individuals with stroke predominantly experience mutual interference, or gait interference without cognitive interference, or gait interference with a reciprocal cognitive benefit (cognitive-priority trade off) (Plummer et al., 2013). To evaluate changes in dual-task interference over time, we propose that researchers and clinicians evaluate the respective gait and cognitive DTE against each other to determine changes in the pattern of interference as well as DTE of each task individually. Then, assuming the rehabilitation goal is to reduce dual-task interference in gait, for patients with mutual interference or gait interference only we propose that movement towards the “no interference” region represents an improvement in dual-task interference. That is, to be considered an improvement there should be a reduction in gait interference without a concurrent increase (worsening) in cognitive interference. If cognitive interference gets worse, this implies that the reduction in gait interference is due to a new trade-off strategy, not an improvement in dual-task gait performance. Similarly, if the treatment focus is to reduce dual-task interference in cognition, cognitive dual-task performance improvement is demonstrated by reduced dual-task costs on the cognitive task only when gait interference also improves or does not change. In a person with mutual interference, dual-task gait improvement can occur as a result of reduced dual-task costs in either one (Figure 2A) or both (Figure 2B) of the tasks. For patients demonstrating a cognitive-priority trade off at baseline, an improvement in dual-task gait performance would still be considered one in which gait interference is reduced, provided cognitive-task performance does not change. For example, as shown in Figure 2C, at baseline the patient demonstrated a dual-task cost on gait but a dual-task benefit on cognition. After treatment, there was no DTE on gait, but the dual-task facilitation on cognitive-task performance remained. This suggests that the allocation of attention to the cognitive task no longer came at a cost to gait, which could be considered a positive outcome.

Evaluating changes in dual-task interference in this way therefore provides insight into changes in attention allocation as well as overall dual-task capacity. This is valuable, because the ability to change the attention allocation strategy in different dual-task situations is likely a highly critical aspect of dual-task performance to ensure safety during community mobility. That is, do patients prioritize attention toward the “right task” at the “right time” to optimize safety? The framework presented here provides clinicians with a tool to be able to analyze how the dual-task interference pattern changes over time and in different dual-task situations, as well as identify when apparent improvements in one task are due to a different attentional strategy rather than overall improvement (Figure 2D). As shown in Figure 2D, although the relative dual-task cost on gait reduced after the intervention, the relative dual-task cost on cognition increased; thus, mutual interference persisted, with a different allocation of capacity/prioritization between the two tasks relative to baseline. In this case, overall dual-task interference has not improved. Reasons for why attention was prioritized differently should be considered. We discuss some potential factors influencing task prioritization later.

While the method for the evaluation of dual-task interference proposed above provides valuable information not otherwise gathered from the traditional approach, it is also not sufficient alone. Absolute measures of dual-task performance should be considered (i.e., actual gait speed in single-task and dual-task conditions) in addition to relative measures of dual-task performance (i.e., DTE%). It has been argued that improvement across all absolute and relative measures of dual-task performance is needed to conclude improvement in overall dual-task performance (Agmon et al., 2015). While we agree with this in principal, there is one caveat: relative dual-task measures may be unimportant at particular values of absolute measures. To illustrate with an example, it is widely accepted that 0.8 m/s is a clinical important threshold for gait speed, because it is the minimum gait speed required for functional community ambulation (Perry et al., 1995). Before intervention, a patient may demonstrate a DTE on gait speed such that dual-task gait speed is below the critical threshold of 0.8 m/s. If the intervention produces increases in both single-task and dual-task gait speed, then the relative measure of dual-task interference (the percent change in dual-task relative to single-task) may not change. However, if the absolute dual-task gait speed now exceeds 0.8 m/s, the relative DTE, although unchanged from baseline, is no longer clinically important. As we emphasized for assessment of relative measures of dual-task interference, changes in absolute measures in both tasks should be considered in concert to fully understand the dual-task performance and determine potential tradeoffs.

## Challenges and Further Considerations for Dual-Task Assessment

The framework and method presented above will help clinicians and researchers better understand dual-task performance and evaluate how performance is changing over time. A current challenge for incorporating this assessment framework into clinical practice is being able to accurately and sensitively measure cognitive performance during dual-task assessment. Two commonly used tasks are accuracy of counting backwards and verbal fluency (e.g., naming words in a particular category). These are not ideal tasks, however, as individuals can slow their responses to maintain accuracy during the dual-task condition, thus potential dual-task performance changes are not easily captured unless response rate is also measured. Other suggestions could include an auditory target detection task or n-back task using taped stimuli to maintain delivery rate. Accuracy of targets detected could then be monitored through verbal responses. Alternatively, a task that can be assessed quantitatively at the end of a walking trial (e.g., a memory task) would also be appropriate. Development of common tasks that are pragmatic yet reliable and sensitive measures of dual-task performance for use across clinical settings would be useful.

There are several other influences on the magnitude and pattern of dual-task interference that need to be considered when implementing and interpreting dual-task assessments. As discussed earlier, when processing capacity is insufficient for task demands, the allocation of capacity will be determined voluntarily or by task-related factors (Tombu and Jolicoeur, 2003; Yogev-Seligmann et al., 2012). Task factors include the nature and difficulty of the gait and cognitive tasks, the presence of other distractions in the environment that may capture (distract) the patient’s attention, and the instructions given to the patient. Patient-related factors include motor and cognitive abilities (including changes in these factors over time, such as in progressive conditions), lesion location, balance confidence, and perceived importance of each task. All of these factors have the potential to influence how one will select to allocate attention in the absence of sufficient processing capacity. The implications for clinical practice are that clinicians need to use the same dual-task combination for pre and post assessments, use a consistent set of instructions, and consider testing more than one dual-task combination.

## Summary Recommendations

To summarize, dual-task interference is the result of the interaction between two simultaneously-performed tasks. Therefore, to accurately interpret dual-task interference, both tasks must be assessed in single-task and dual-task conditions. The following specific recommendations are provided for clinicians and researchers:

1. Establish a standardized assessment protocol for dual-task assessment; use consistent instructions.
2. Measure key parameters of performance for both tasks in single-task and dual-task conditions.
3. Examine changes in absolute and relative measures of both tasks.
4. Evaluate dual-task changes in one task in relation to the other task to gather information about the attentional strategy and potential tradeoffs.
5. Assess treatment-related changes in absolute measures against known MCID values or other known clinically significant thresholds to determine if changes are meaningful.
6. Evaluate treatment-related changes in relative measures in terms of pattern/strategy change; be mindful of how changes in non-treatment factors may have contributed to changes in performance over time (e.g., progressive conditions).

The methods and recommendations proposed in this paper will help advance the theoretical framework for neurorehabilitation related to mobility recovery in individuals with neurological disorders by ensuring that the interaction between simultaneously-performed tasks is taken into account when evaluating dual-task performance. The proposed conceptual framework for classifying dual-task interference and evaluating the effects of rehabilitation also serves to bridge two translational road blocks: translation of theoretical principles and knowledge into new approaches for examination and treatment, and translation of research into clinical practice. The relevance of dual-task walking to everyday ambulation is difficult to dispute, and numerous studies have established the negative impact that dual-task interference can have on gait in people with neurological disorders and older adults (Al-Yahya et al., 2011). To date, interventions targeting gait-related dual-task interference show some promising but mixed results (Brauer and Morris, 2010; An et al., 2014; Peirone et al., 2014; Plummer et al., 2014, 2015). Improved understanding of the mechanisms and behavior underlying dual-tasking may lead to development of more specific treatment approaches or activities. The model proposed here will enable clinicians and researchers to identify underlying attentional strategies or patient preferences in particular dual-task situations, which may help inform treatment decisions and ultimately improve functional mobility in people with neurological disorders.

## Conflict of Interest Statement

The authors declare that the research was conducted in the absence of any commercial or financial relationships that could be construed as a potential conflict of interest.
